# Diagnostic criteria for erosive lichen planus affecting the vulva: an international electronic-Delphi consensus exercise

**DOI:** 10.1111/bjd.12334

**Published:** 2013-08-13

**Authors:** RC Simpson, KS Thomas, P Leighton, R Murphy

**Affiliations:** 1 Centre of Evidence Based Dermatology, University of NottinghamKing's Meadow Campus, Lenton Lane, Nottingham, NG7 2NR, U.K; 2 Division of Primary Care, University of NottinghamKing's Meadow Campus, Lenton Lane, Nottingham, NG7 2NR, U.K; 3 Department of Dermatology, Nottingham University HospitalsNottingham, U.K

## Abstract

Background There is no defined set of criteria for diagnosing erosive lichen planus affecting the vulva (ELPV) and there is geographical variation in management.

Objectives To reach consensus on clinicopathological diagnostic criteria for ELPV.

Methods This was a three-stage international electronic-Delphi exercise with a subsequent formal feedback process. In the first two rounds participants were asked to rate the importance of a list of clinicopathological criteria. Responses from round 1 were summarized and presented in round 2, along with additional criteria suggested by participants. In round 3, participants were asked to rate the items that had reached consensus as ‘essential’ or ‘supportive’ features in diagnosing ELPV. Consensus was defined as being reached if 75% of participants agreed on the importance of an item.

Results A total of 73 experts representing dermatology, gynaecology, histopathology and genitourinary medicine participated; 69 (95%) completed all three rounds. Consensus was achieved for the following ‘supportive’ diagnostic criteria: (i) well-demarcated erosions/erythematous areas at the vaginal introitus; (ii) presence of a hyperkeratotic border to lesions and/or Wickham striae in surrounding skin; (iii) symptoms of pain/burning; (iv) scarring/loss of normal architecture; (v) presence of vaginal inflammation; (vi) involvement of other mucosal surfaces; (vii) presence of a well-defined inflammatory band involving the dermoepidermo junction; (viii) presence of an inflammatory band consisting predominantly of lymphocytes; and (ix) signs of basal layer degeneration. It was suggested that at least three supportive features should be present to make a diagnosis of ELPV, although this number is subject to further discussion.

Conclusions This study has identified a diagnostic dataset for ELPV that can be adopted into clinical practice and clinical trials.

## What's already known about this topic?

- Erosive lichen planus affecting the vulva (ELPV) is an uncommon inflammatory dermatosis that is often resistant to first-line therapy.
- There are no published criteria for the diagnosis of ELPV.

## What does this study add?

- Using the electronic-Delphi technique we have collated a set of nine diagnostic criteria internationally agreed by physicians with expertise in the diagnosis and management of vulval disease including ELPV.
- It is thought that at least three out of the nine supportive criteria should be present in order to diagnose ELPV, but this number requires further validation.
- This diagnostic dataset will guide the clinical diagnosis of ELPV and will standardize the inclusion of patients into clinical trials.

Erosive lichen planus (ELP) is a chronic, T-cell-mediated inflammatory condition affecting the squamous epithelium. It involves mucocutaneous sites, particularly the orogenital mucosa. Most clinicians managing vulvar diseases would diagnose ELP affecting the vulva (ELPV) following careful clinicopathological correlation.^1^ However, unlike oral lichen planus, which has a defined set of diagnostic criteria as set out by the World Health Organization in 1978^2^ and subsequently modified in 2003, the same does not exist for vulval disease.

ELPV may mimic other conditions such as lichen sclerosus (with which it may overlap clinically and histopathologically),^3^,^4^ autoimmune bullous disorders and intraepithelial carcinoma.^4^ The diagnosis can therefore be challenging.

Early recognition of vulvovaginal lichen planus is important to minimize unnecessary medical or surgical procedures and to instigate prompt treatment and alleviation of symptoms.^6^–^7^ However, ELPV may present to a range of specialties such as general dermatology, gynaecology and genitourinary medicine, where variation in diagnosis and management exists.^8^

An agreed diagnostic dataset would be valuable to standardize practice, to assist nonexperts in making a correct diagnosis and to regulate inclusion into clinical trials. The purpose of this international, multiperspective, electronic-Delphi (e-Delphi) consensus exercise was to reach agreement on a diagnostic dataset for ELPV that is acceptable to the international clinical community.

## Methods

### Study type

This was a three-stage, international e-Delphi exercise that was conducted between October 2012 and December 2012. The Delphi process is widely used in clinical and health services research;^9^ it is an iterative technique based on the scoring of a series of structured statements that are revised and repeated until consensus has been reached among a panel of expert participants.^10^ It is a method frequently used for establishing diagnostic criteria.^11^–^12^ We conducted this study as an electronic process and the exercise was moderated by a single central coordinator (R.C.S.).

### Participants

A letter of invitation was emailed to all members of the International Society for the Study of Vulvovaginal Disease (ISSVD) and members of the British Society for the Study of Vulval Disease (BSSVD). These are multidisciplinary societies comprising experts from different stakeholder groups who manage patients with vulvovaginal disease. Members of these societies were identified as potential participants of this e-Delphi study as they are professionals with a specialist interest in the relevant field, they will directly utilize the outcomes of the e-Delphi study in their daily practice and they have the skill set to make an insightful, well-informed contribution to the exercise.

No inconvenience allowance was offered and response to the initial invitation was taken as implied consent to participate in the study. Ethical approval was not required as all participants were healthcare professionals who participated as part of their professional role.

### Study procedures

To provide an evidence base for the consensus exercise, a review of the literature was undertaken to summarize diagnostic criteria that have been used in previous studies of ELPV. In addition, items perceived important by 25 clinicians were elicited through a set of structured interviews.^13^ The results from these two exercises were collated to form a structured questionnaire that contained a list of 12 potential diagnostic criteria required for the diagnosis of ELPV. The study protocol was finalized in September 2012.

The exercise was conducted anonymously, except for the coordinator, who was required to know participants' details for administrative purposes. Participants were asked specifically for their consent to be acknowledged in future presentation or publication.

Questionnaires were completed using the online ‘SurveyMonkey’ tool.^14^ A 2-week period for each round was given in which participants could submit their responses.^15^ Reminders for each round were sent at 7, 10 and 14 days to nonresponders.

In the first round of the e-Delphi exercise, participants were asked to rate the importance of the selected 12 diagnostic criteria on a five-point Likert scale (‘very important’, ‘important’, ‘less important’, ‘not important’ and ‘not sure’). When discussing histological criteria, it was specified that biopsy samples should be taken from the edge of an erosion where representative histology would most likely be present. Contributors were asked to list any additional diagnostic features not in the original list that they considered relevant. The survey instrument was amended following round 1. Diagnostic items for which consensus was reached as ‘not important’ were removed and additional diagnostic items were incorporated into the questionnaire.

In the second round summary scores for round 1 were presented and respondents could submit new answers or leave their original responses unchanged. The same process of analysis and amendment of the survey tool occurred to create the round 3 questionnaire.

In the third round participants were asked to rate criteria that had reached consensus as important as ‘essential’, ‘supportive’ or ‘neither’. ‘Essential’ was defined as a diagnostic feature that must be present to make a diagnosis of ELPV. ‘Supportive’ was classed as a feature that does not have to be present, but adds weight to other diagnostic features that are present. Participants were also asked how many essential and/or supportive diagnostic criteria should be present to make a diagnosis of ELPV.

It was made clear throughout all rounds if questions had been amended, added or excluded following analysis of previous rounds. Participants were given the opportunity to comment on any of these amendments. After completion and analysis of all three rounds, the findings were circulated for formal feedback and comments from the participants.

### Definition of consensus

Consensus was defined as being reached if 75% of participants agreed on the importance of an item, i.e. rated it ‘very important’ or ‘important’ on the Likert scale, or agreed whether an item should be ‘essential’ or ‘supportive’. As a soft measure of consensus to avoid premature exclusion of diagnostic items, we also carried through items that less than 25% of participants had rated ‘not important’ or ‘unsure’. Diagnostic criteria that did not achieve consensus/soft consensus were excluded from subsequent rounds of the exercise.

## Results

The letter of invitation was circulated to 283 members of the ISSVD and 175 members of the BSSVD. Some physicians were members of both societies but for confidentiality reasons these data are unknown. A total of 73 individuals participated in the first round. Of these, 71 (97%) completed the second round and 69 (95%) completed the final round. The formal feedback survey was completed by 54 participants.

Participants represented four distinct stakeholder groups and were from 14 different countries. The majority had over 10 years' experience in managing patients with vulval skin disease and 88% of respondents were either professors or consultants in their field. Characteristics of the participants are shown in Table 1.

**Table 1:** Characteristics of participants in the electronic-Delphi exercise

	Round 1 participants, *n* (%)	Round 2 participants, *n* (%)	Round 3 participants, *n* (%)
Total participants in each round	73	71	69
Stakeholder group			
Dermatology	30 (41)	30 (42)	30 (43)
Gynaecology (± obstetrics)	30 (41)	28 (39)	26 (38)
Histopathology/dermatopathology	7 (10)	7 (10)	7 (10)
Genitourinary medicine/venerology	6 (8)	6 (8)	6 (9)
Grade			
Professor/associate professor	19 (26)	18 (25)	17 (25)
Consultant	45 (62)	45 (63)	45 (65)
Associate specialist	6 (8)	5 (7)	4 (6)
Resident/specialist registrar	2 (3)	2 (3)	2 (3)
Specialist nurse	1 (1)	1 (1)	1 (1)
Country			
Argentina	2	2	2
Australia	7	7	7
Canada	3	3	3
Denmark	1	1	1
France	2	2	1
Germany	1	1	1
Israel	1	1	1
Italy	2	2	2
Netherlands	3	3	3
New Zealand	1	1	1
Portugal	1	1	1
U.K.	34	33	33
Uruguay	1	1	1
U.S.A.	14	13	12
Duration of experience			
< 5 years	11 (15)	8 (11)	7 (10)
6–10 years	12 (16)	13 (18)	12 (17)
11–15 years	15 (21)	15 (21)	14 (20)
16–20 years	18 (25)	18 (25)	22 (32)
> 20 years	17 (23)	17 (24)	14 (20)

Following the first round, two clinical and three histopathological items were added to the round 2 questionnaire. Additionally, the wording of four questions was amended for clarity.

Six potential diagnostic criteria were removed following rounds 1 and 2 as participants' answers indicated these were not important to diagnose ELPV (Table 2).

**Table 2:** Diagnostic criteria excluded after first and second Delphi rounds (> 25% participants considered these ‘not important’ or ‘not sure’)

Diagnostic item	Responses, *n* (%)					
	Very important	Important	Less important	Not important	Not sure
Excluded after round 1					
Presence of symmetrical lesions	2 (3)	9 (12)	30 (41)	30 (41)	2 (3)
Presence of vaginal discharge	1 (1)	10 (14)	30 (41)	30 (41)	2 (3)
Presence of pain on Q-tip pressure	2 (3)	8 (11)	21 (29)	38 (52)	4 (5)
Excluded after round 2					
Findings on wet mount preparation	2 (3)	5 (7)	27 (38)	28 (39)	9 (13)
Presence of epidermal changes on histopathological examination	5 (7)	20 (28)	25 (35)	8 (11)	13 (18)
Direct immunofluorescence	3 (4)	12 (17)	29 (41)	20 (28)	7 (10)

Ten diagnostic features (six clinical and four histopathological) reached consensus, or soft consensus, and were carried through to the third round for final approval (Table 3).

**Table 3:** Round 2 results. Items that reached consensus as important (i.e. where > 75% participants rated ‘very important’ or ‘important’; in normal text) were carried through into the final round. Items that did not meet this cut-off, but where < 25% participants rated ‘not important’ or ‘not sure’, were also carried through as a measure of ‘soft consensus’ (marked in italics). Items that were dropped following round 2 are in bold

Diagnostic item	Responses, *n* (%)					
	Very important	Important	Less important	Not important	Not sure
Clinical					
Presence of well-demarcated erosions or glazed erythema at the vaginal introitus	41 (58)	26 (37)	0 (0)	1 (1)	3 (4)
Scarring/loss of normal architecture	13 (18)	46 (65)	10 (14)	0 (0)	2 (3)
*Presence of a hyperkeratotic white border to erythematous areas/erosions ± Wickham striae in surrounding skin*	*9 (13)*	*37 (52)*	*21 (30)*	*2 (3)*	*2 (3)*
*Presence of vaginal inflammation ± vaginal scarring*	*7 (10)*	*20 (28)*	*34 (48)*	*8 (11)*	*2 (2·83)*
*Involvement of other mucosal sites, e.g. mouth, oesophagus*	*13 (18)*	*31 (44)*	*21 (30)*	*4 (6)*	*2 (3)*
*Symptoms of pain/burning*	*16 (22·53)*	*32 (45)*	*18 (25)*	*3 (4)*	*2 (3)*
**Findings on wet mount preparation**	**2 (3)**	**5 (7)**	**27 (38)**	**28 (39)**	**9 (13)**
Histopathological					
Presence of a well-defined inflammatory band in the superficial connective tissue that involves the dermoepidermal junction	27 (38)	40 (56)	3 (4)	0 (0)	1 (1)
Presence of an inflammatory band that consists predominantly of lymphocytes	6 (8)	60 (85)	3 (4)	0 (0)	2 (3)
Signs of basal cell layer degeneration, e.g. Civatte bodies, abnormal keratinocytes or basal apoptosis	13 (18)	47 (66)	7 (10)	0 (0)	4 (6)
*Absence of dermal hyalinization*	*8 (11)*	*17 (24)*	*29 (41)*	*3 (4)*	*14 (20)*
**Epidermal changes, e.g. wedge-shaped hypergranulosis, saw-toothed acanthosis**	**5 (7)**	**20 (28)**	**25 (35)**	**8 (11)**	**13 (18)**
**Findings on direct immunofluorescence**	**3 (4)**	**12 (17)**	**29 (41)**	**20 (28)**	**7 (10)**

In the third and final round, participants were asked to rank items as ‘essential’ or ‘supportive’ diagnostic criteria, or neither (Table 4). No diagnostic indicator reached consensus as being ‘essential’. The ‘absence of dermal hyalinization’ on histopathological examination was not favoured as being in the final dataset. The remaining nine diagnostic items were recommended as being supportive diagnostic criteria (Table 4); the resulting dataset therefore consisted of nine criteria that represent clinicopathological features of ELP. Of the 54 participants who provided feedback 93% were in agreement with this.

**Table 4:** Round 3 results; essential and supportive diagnostic criteria and final diagnostic dataset. The item that did not reach consensus and was subsequently removed is in bold

Diagnostic item	Responses, n (%)			
	Essential	Supportive	Neither
Presence of well-demarcated erosions or glazed erythema at the vaginal introitus	44 (64)	24 (35)	1 (1)
Presence of a hyperkeratotic white border to erythematous areas/erosions ± Wickham's striae in surrounding skin	8 (12)	57 (83)	4 (6)
Symptoms of pain/burning	13 (19)	47 (68)	9 (13)
Scarring/loss of normal architecture	10 (14)	55 (80)	4 (6)
Presence of vaginal inflammation	7 (10)	48 (70)	14 (20)
Involvement of other mucosal sites	1 (1)	66 (96)	2 (3)
Presence of a well-defined inflammatory band in the superficial connective tissue that involves the dermo-epidermo junction	37 (54)	32 (46)	0 (0)
Presence of an inflammatory band that consists predominantly of lymphocytes	30 (43)	37 (54)	2 (3)
Signs of basal cell layer degeneration, e.g. Civatte bodies, abnormal keratinocytes or basal apoptosis	24 (35)	43 (62)	2 (3)
**Absence of dermal hyalinization**	11 (16)	38 (55)	20 (29)

When asked in round 3 how many supportive features should be present to diagnose ELPV, consensus was reached for at least three out of nine needing to be present. However, following participant feedback, opinion was divided between three or four supportive features being required.

During the exercise, participants were asked about the importance of performing diagnostic biopsy. There was disparity in opinion, with 36/69 (52%) responding that a diagnosis of ELPV does *not* always have to satisfy clinical and histopathological criteria. However, 63/69 (91%) acknowledged that a biopsy should be performed if there was diagnostic uncertainty or concern of neoplastic change. The differential diagnoses identified as most likely to cause diagnostic difficulty were lichen sclerosus and mucosal autoimmune bullous disorders.

## Discussion

This exercise enabled the collation of a set of nine diagnostic criteria defined by experts as supportive of the diagnosis of ELPV (Table 5); no essential features were identified. It was agreed that three or more of these supportive features are required to diagnose ELPV and these can be a combination of both histological and clinical features. However, feedback from participants suggested that more focused work is required to determine whether this is the optimum number of features and whether the individual items should be weighted.

**Table 5:** Final diagnostic dataset. Diagnosis of erosive lichen planus affecting the vulva requires three out of the nine criteria listed here

	Criteria
1	Presence of well-demarcated erosions or glazed erythema at the vaginal introitus
2	Presence of a hyperkeratotic white border to erythematous areas/erosions ± Wickham striae in surrounding skin
3	Symptoms of pain/burning
4	Scarring/loss of normal architecture
5	Presence of vaginal inflammation
6	Involvement of other mucosal sites
7	Presence of a well-defined inflammatory band in the superficial connective tissue that involves the dermoepidermal junction
8	Presence of an inflammatory band that consists predominantly of lymphocytes
9	Signs of basal cell layer degeneration, e.g. Civatte bodies, abnormal keratinocytes or basal apoptosis

The e-Delphi method was used to answer a research question that required specialist input from the clinical community as these data were not available in the existing literature. The Delphi technique is characterized by four core features: the involvement of an expert panel, multiple iterations, feedback between rounds and anonymity.^16^ The latter is particularly important as in face-to-face group-based processes the presence of dominant individuals can have a large influence on the results.^15^ Each of these core features was embodied by this study.

Due to the study conduct being via web-based communication, geographical constraints were overcome and anonymity of participants was maintained. There was a high degree of experience and skill within the recruited group. All participants were members of specialist societies with a specific interest in vulvovaginal disease. The demographics of the group indicate that respondents had the necessary skills and experience to contribute to the derived diagnostic dataset.

We ran three rounds of the Delphi exercise, which enabled the study to be completed in a timely manner without participants developing survey ‘fatigue’. Feedback indicated that three rounds were sufficient to formulate a list of clinicopathological features that are suggestive of ELPV, but further work is needed to determine the exact number of these criteria required.

Important considerations when interpreting the results of this exercise are that two of the stakeholder groups, dermatopathology and genitourinary medicine, were under-represented. Reliability of responses from individual groups diminishes with numbers of fewer than 12 and are considered to be unreliable with six or fewer.^10^ While dermatology and gynaecology expertise was adequately represented by respondents (Table 1), histological opinion was not, as only seven dermatopathologists took part. Individual histopathologists did comment that epidermal changes such as sawtoothed acanthosis and hypergranulosis, and dermal changes of lack of hyalinization, were important. These comments were not sufficient to alter the results; however, findings may be different with larger numbers. We do not know if the views of the seven dermatopathologists were representative of the profession as a whole, but it was beyond the scope of this exercise to investigate further.

It was important to do this exercise for two reasons: firstly to improve the diagnosis of an uncommon condition and improve patient care, and secondly to define stringent diagnostic criteria so that robust clinical trials can be carried out to improve current patient management.^8^ This is particularly crucial as patients with ELPV may present to various specialty groups.

Participants agreed that ELPV can be diagnosed clinically and a biopsy does not always need to be taken. However, biopsy should be performed in cases of diagnostic doubt or if there is suspicion of malignancy.

The site of biopsy is important as histological features described in the diagnostic dataset are more likely to be present at the edge of an erosion than centrally. Classical lichenoid features are most likely to be found when taken from the white margin of erosions.^6^ Assessment of vulval biopsies should be by a dermato-or gynaepathologist as changes of lichen planus are often subtle and there is a possibility of an incorrect diagnosis being made by pathologists who are inexperienced in this field.^17^

The interest and high fidelity demonstrated in all three rounds shows that physicians internationally are motivated to advance practice in this area of vulvovaginal disease; 73 experts participated in the first round and only four dropped out during the 9-week study period.

It should be realized that this is just one utility of the Delphi process and the methodology can be translated to other areas of healthcare where information in the scientific literature is lacking and therefore needs to be generated using expert opinion, for example in establishing core outcome sets.^18^

In conclusion, this consensus exercise represents the views of a group of experts and provides a list of supportive features that are considered central to diagnosing ELPV. The next steps are to validate the diagnostic criteria in the clinical setting by applying them to patients managed during normal practice. We envisage that the diagnostic criteria will guide physicians in their daily practice and that future clinical trials in this field will utilize common diagnostic criteria to ensure inclusion of comparable participants.
